# Supplementary material for: A serum microRNA classifier for the diagnosis of sarcomas of various histological subtypes
Source: Nat Commun. 2019 Mar 21;10:1299. doi: 10.1038/s41467-019-09143-8 (PMC6428849; doi:10.1038/s41467-019-09143-8)
Supplement: Supplementary file 2 — Supplementary Information [file 41467_2019_9143_MOESM2_ESM.pdf]

Supplementary Information

A serum microRNA classifier for the diagnosis of sarcomas of various histological subtypes

Asano and Matsuzaki et al.

Supplementary Information

Supplementary Figure 1. Serum miRNA profiles in patients with bone and soft tissue tumors
Supplementary Figure 2. Cluster analysis of 83 identified serum miRNAs in the training cohort
Supplementary Figure 3. Quantitative RT-PCR as orthogonal validation
Supplementary Figure 4. Index VI values according to TNM stage parameters
Supplementary Figure 5. The utility of Index VI as a sarcoma biomarker
Supplementary Figure 6. Correlation between miRNA levels in serum and tissue samples
Supplementary Table 1. Histological diagnosis of participants in the discovery, training, and validation sets
Supplementary Table 2. Histological diagnosis of participants in the exploratory set
Supplementary Table 3. Differences in characteristics between the malignant, benign, healthy, and intermediate groups
Supplementary Table 4. Serum expression levels of selected miRNAs in the discovery set
Supplementary Table 5. Odds ratios for discriminating sarcoma
Supplementary Table 6. Information on the miScript PCR primers
Supplementary Methods

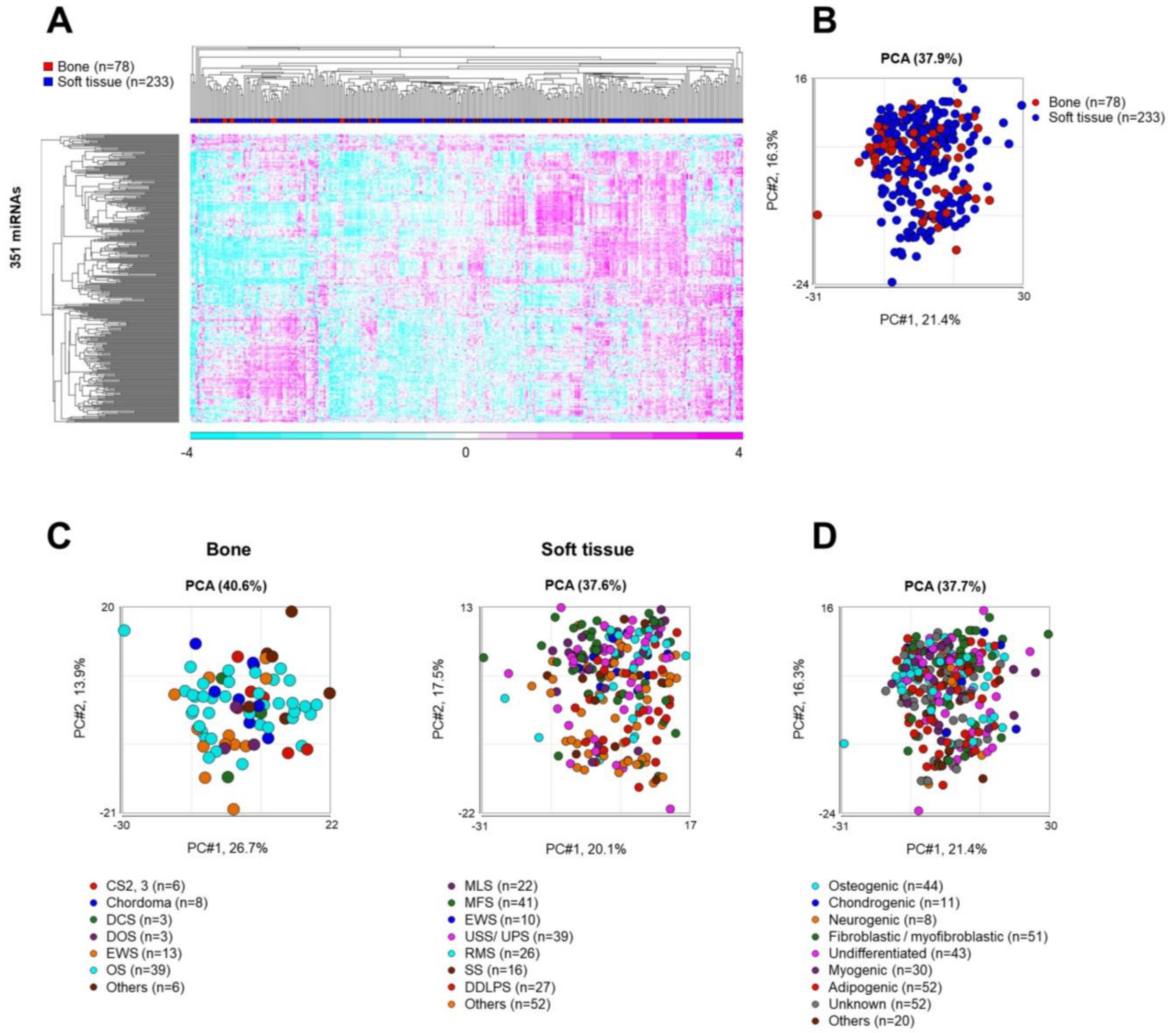

Supplementary Figure 1: Serum miRNA profiles in patients with bone and soft tissue tumors

Serum miRNA profiles were compared between bone tissue and soft tissue tumors by (A) unsupervised cluster analysis and (B) PCA; and among (C) histological subtypes and (E) tumor differentiation lineage by PCA. CS2,3, chondrosarcoma, grades II and III; DOS, differentiated osteosarcoma; DCS, differentiated chondrosarcoma; EWS, Ewing sarcoma; OS, osteosarcoma; MLS, myxoid liposarcoma; MFS, myxofibrosarcoma; USS/UPS, undifferentiated spindle cell sarcoma/undifferentiated pleomorphic sarcoma; RMS, rhabdomyosarcoma; SS, synovial sarcoma; DDLPS, dedifferentiated liposarcoma.

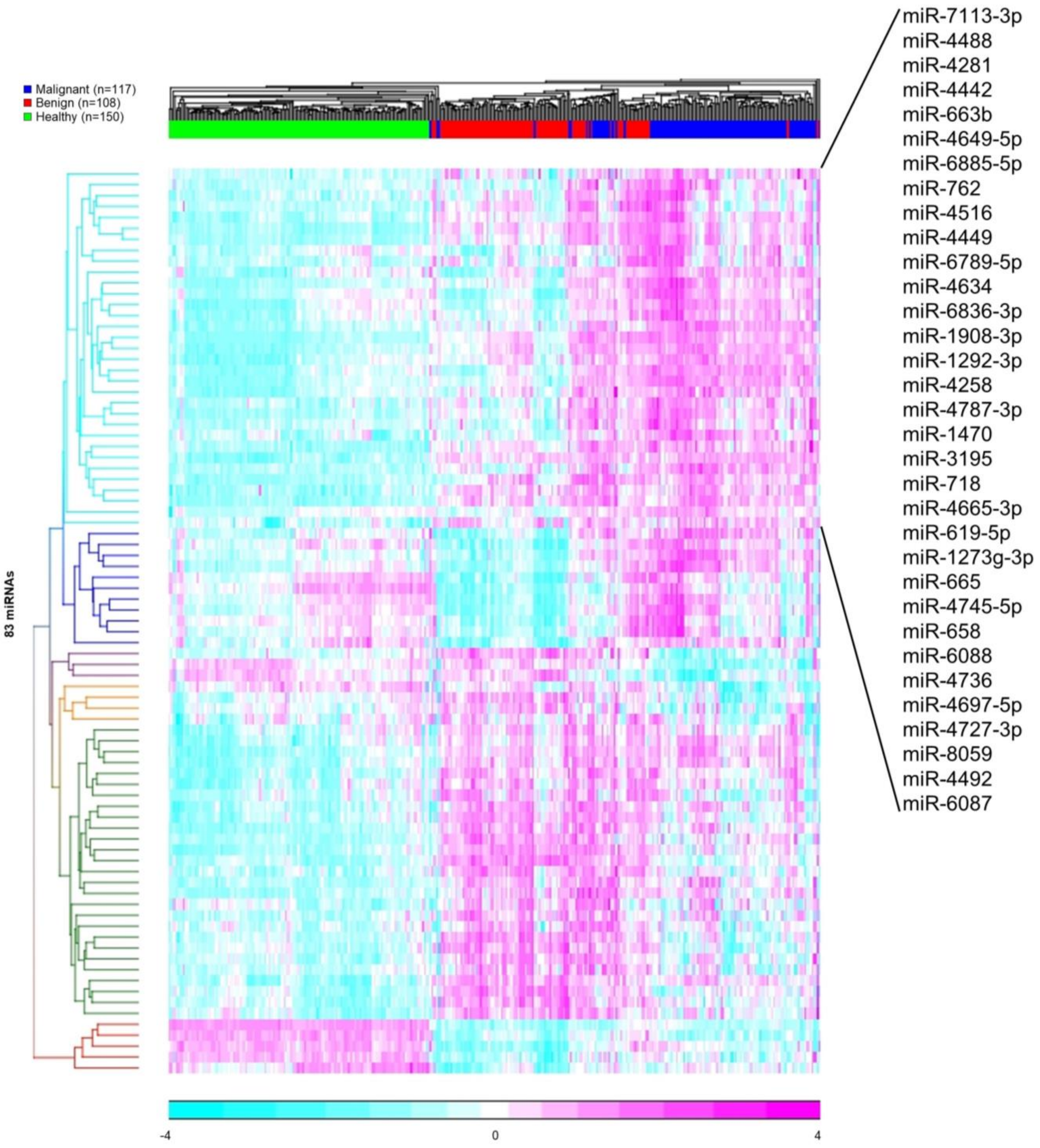

Supplementary Figure 2: Cluster analysis of 83 identified serum miRNAs in the training cohort

83 miRNAs were divided into six clusters. One cluster (shown in sky blue), in which expression was increased through healthy, benign, and malignant groups in a step-by-step manner, included 33 miRNAs. These miRNAs were considered to be biomarker candidates.

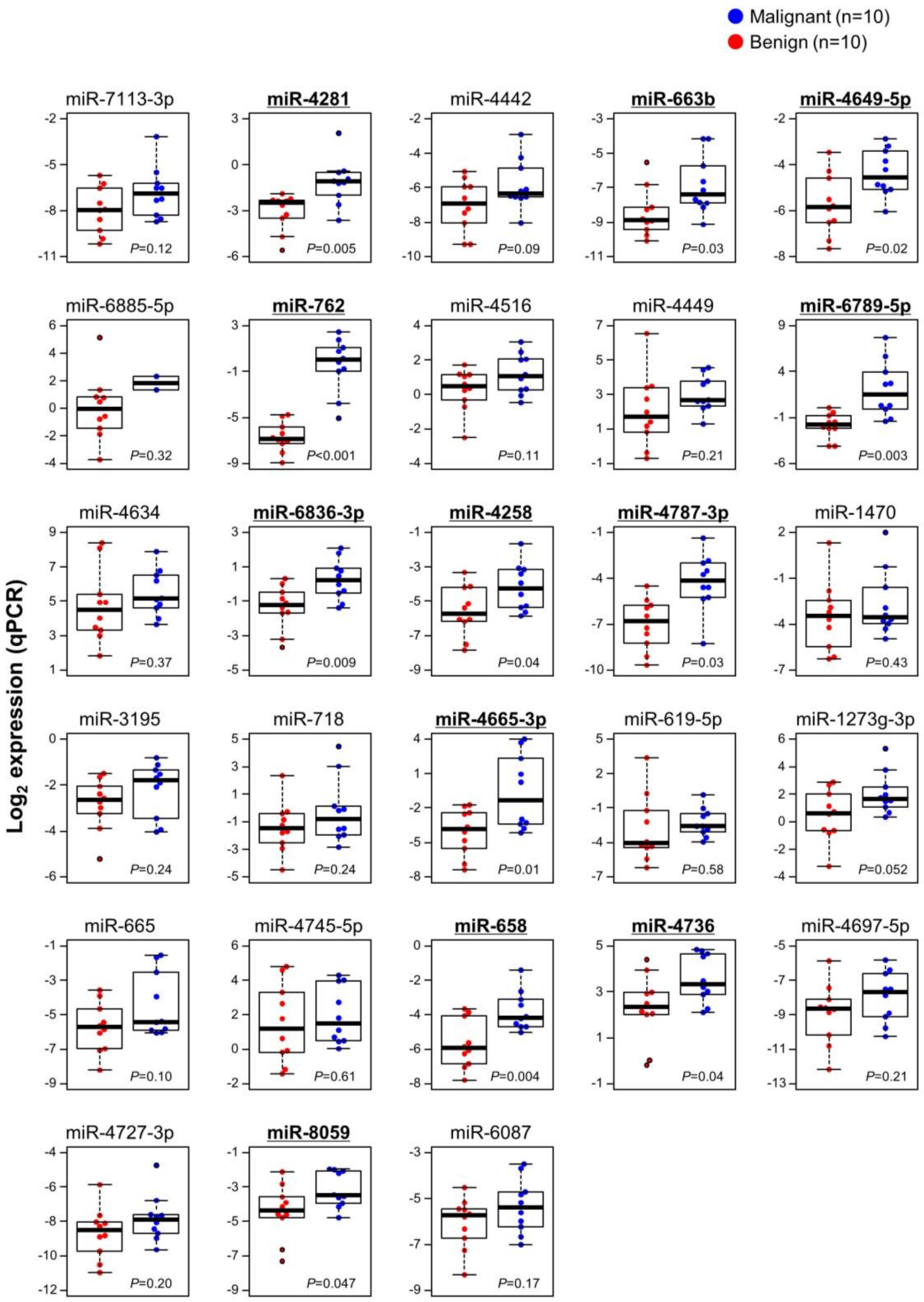

Supplementary Figure 3: Quantitative RT-PCR as orthogonal validation

Serum levels of each miRNA were quantified by qRT-PCR in 10 malignant and 10 benign bone and soft tissue tumor samples. *P* values were calculated by Student's *t*-test. Twelve miRNAs (underlined) that were upregulated in the malignant group ($P < 0.05$) were included in further analyses. Box and whisker plots represent the median, 25th and 75th percentiles, and range of the data.

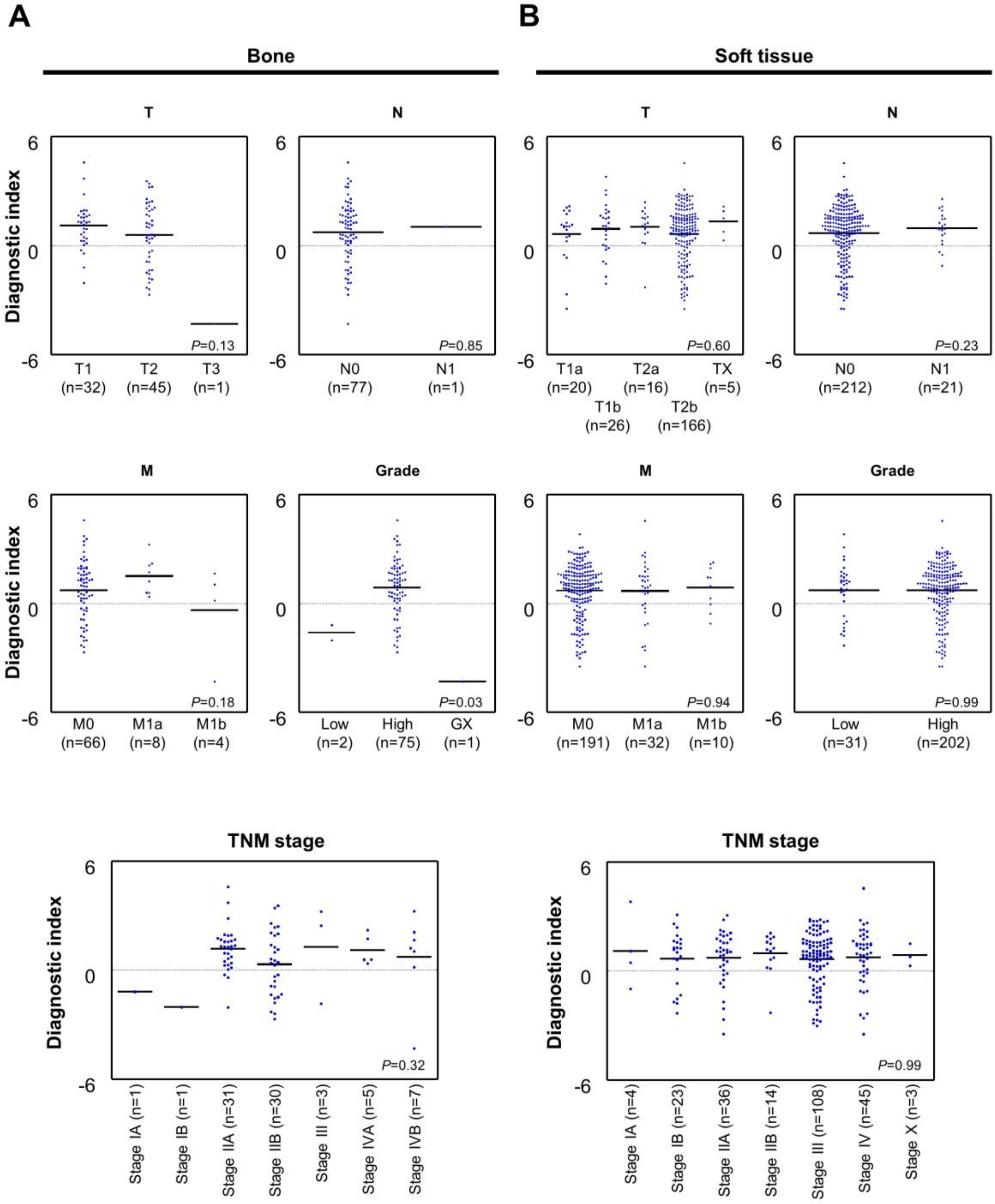

Supplementary Figure 4: Index VI values according to TNM stage parameters

The values of Index VI were compared according to primary tumor size and depth (T), regional lymph node metastasis at present (N), distant metastasis at present (M), histological grade (Grade), and TNM stage in bone sarcomas (A) and soft tissue sarcomas (B) in the discovery, training, and validation sets. In bone sarcomas: T1, ≤8 cm; T2, >8 cm; T3, discontinuous tumors; N0, no regional lymph node metastasis; N1, regional lymph node metastasis; M0, no distant metastasis; M1, distant metastasis. In soft tissue sarcomas: T1a, ≤5 cm and superficial tumor; T1b, ≤5 cm and deep tumor; T2a, >5 cm and superficial tumor; T2b, >5 cm and deep tumors; TX, primary tumor cannot be assessed.

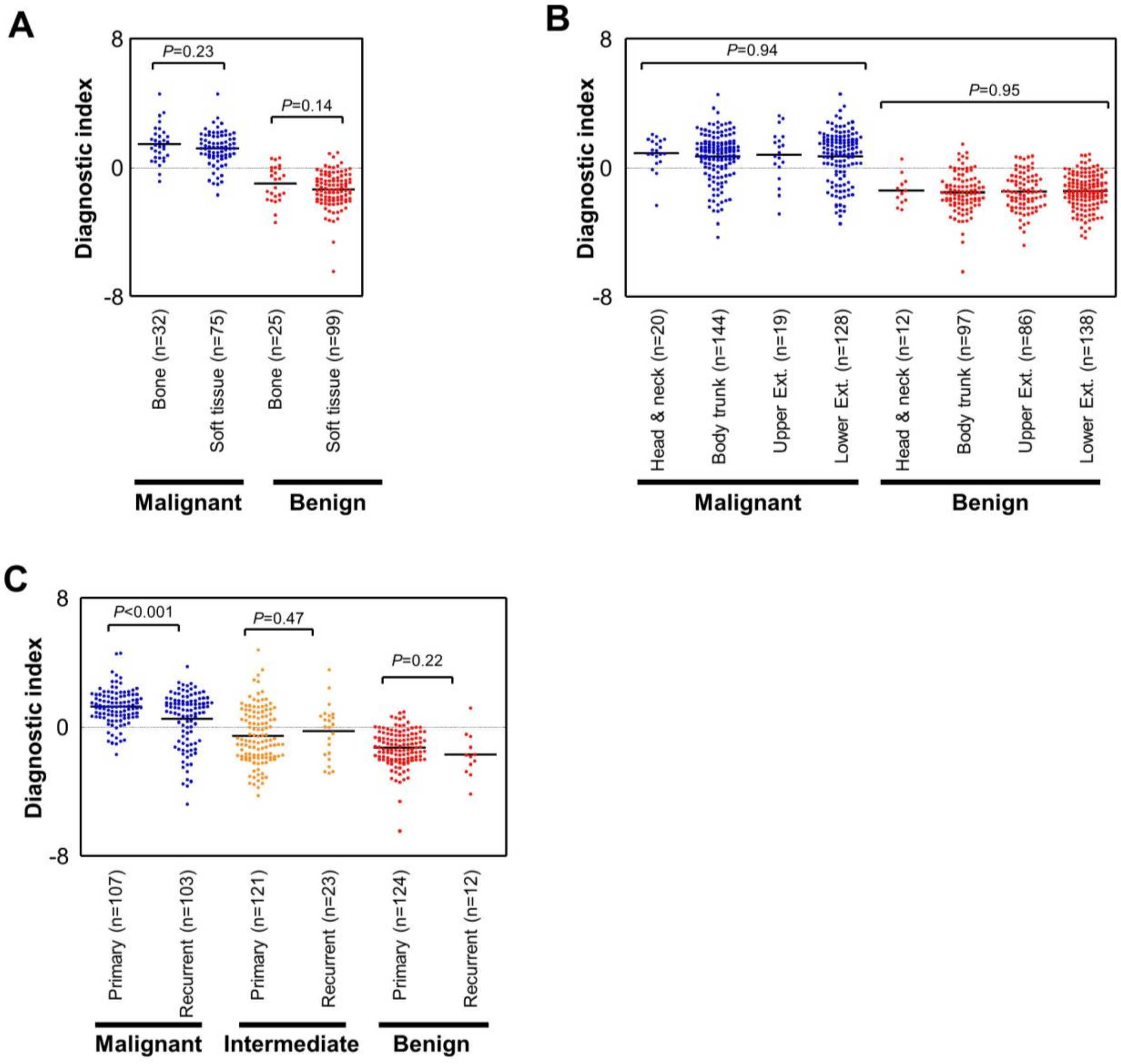

Supplementary Figure 5: The utility of Index VI as a sarcoma biomarker

The values of Index VI were compared according to (A) tumor tissue origin and (B) tumor site in the validation set, and (C) initial tumor status (primary vs. recurrent) in the validation and exploratory sets.

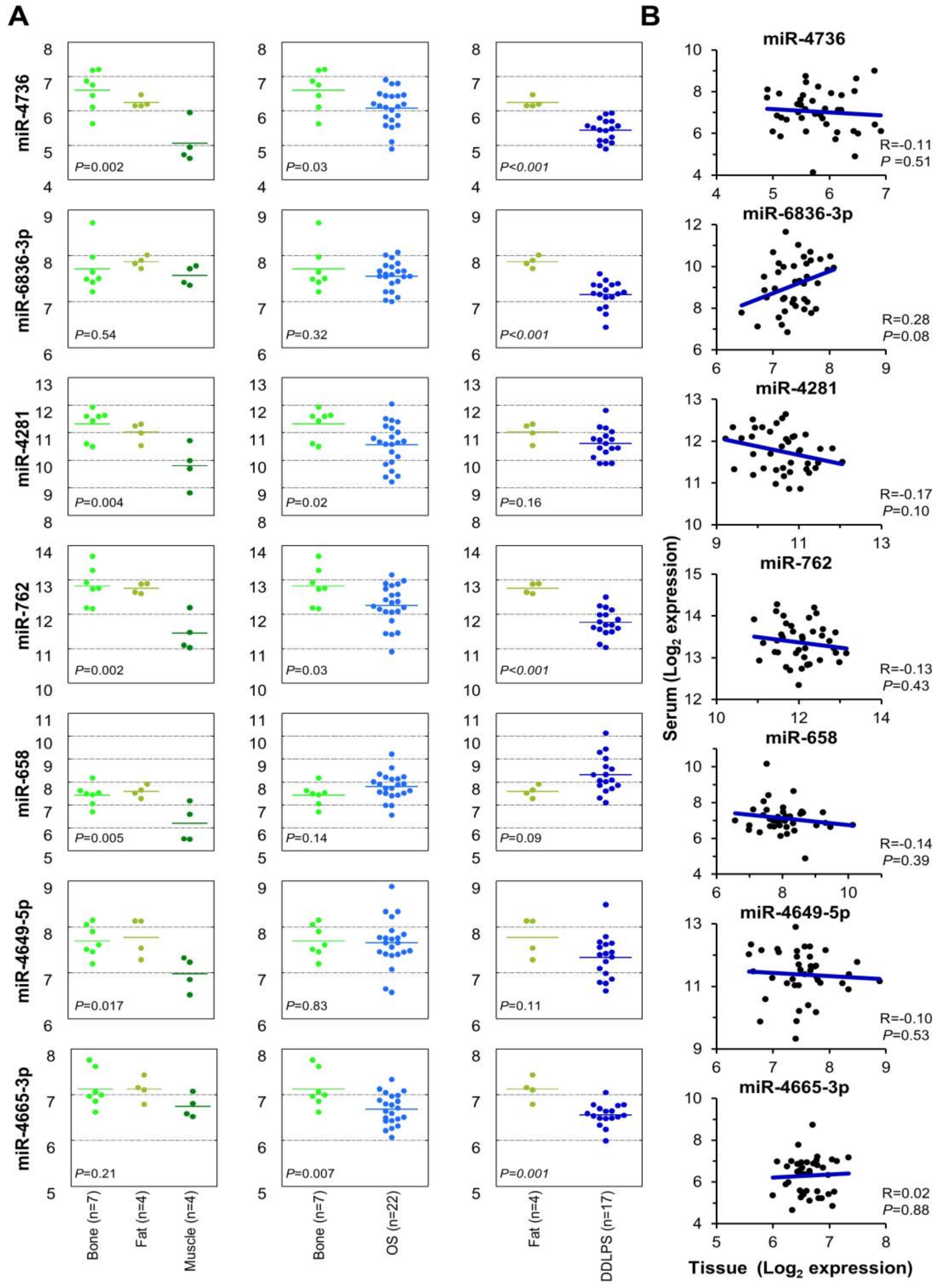

Supplementary Figure 6: Correlation between miRNA levels in serum and tissue samples

(A) Expression levels of the seven miRNAs included in Index VI, as determined by microarray analysis in normal bone (n=7), fat (n=4), muscle (n=4), and tumor tissues (22 osteosarcomas and 18 dedifferentiated liposarcomas). (B) Correlation analyses of the expression levels of the seven miRNAs included in Index VI, comparing serum vs. tissue samples in 40 patients with sarcoma (22 osteosarcomas and 18 dedifferentiated liposarcomas). R = Spearman's correlation coefficient.

Supplementary Table 1: Histological diagnosis of participants in the discovery, training, and validation sets

	Discovery set	Training set	Training set 2	Validation set	*P* value[a]
Malignant					
Bone	n=20	n=23	n=3	n=32	0.83
Dedifferentiated osteosarcoma	0 (0%)	1 (4.3%)	0 (0%)	2 (6.3%)	
Dedifferentiated chondrosarcoma	0 (0%)	1 (4.3%)	0 (0%)	2 (6.3%)	
Chondrosarcoma, grades II, III	1 (5.0%)	3 (13.0%)	0 (0%)	2 (6.3%)	
Ewing sarcoma	2 (10.0%)	4 (17.4%)	1 (20.0%)	6 (18.8%)	
Osteosarcoma	11 (55.0%)	12 (52.2%)	2 (45.7%)	14 (43.8%)	
Chordoma	2 (10.0%)	2 (8.7%)	0 (0%)	4 (12.5%)	
Others	4 (20.0%) [b]	0 (0%)	0 (0%)	2 (6.3%)	
Soft tissue	n=57	n=94	n=7	n=75	0.012
Ewing sarcoma	0 (0%)	7 (7.4%)	0 (0%)	3 (4.0%)	
Myxofibrosarcoma	4 (7.0%)	**24 (25.5%)**	1 (14.3%)	12 (16.0%)	
USS/UPS	9 (15.8%)	13 (13.8%)	**4 (57.1%)**	13 (17.3%)	
Myxoid liposarcoma	3 (5.3%)	9 (9.6%)	1 (14.3%)	9 (12.0%)	
Synovial sarcoma	8 (14.0%)	5 (5.3%)	0 (0%)	3 (4.0%)	
Rhabdomyosarcoma	7 (12.3%)	10 (10.6%)	1 (14.3%)	8 (10.7%)	
Dedifferentiated liposarcoma	5 (8.8%)	10 (10.6%)	0 (0%)	12 (16.0%)	
Others	**21 (36.8%)**	16 (17.0%)	0 (0%)	15 (20.0%)	
Benign					
Bone	n=15	n=23	n=6	n=25	0.95
Inflammation	2 (13.3%)	2 (8.7%)	1 (16.7%)	1 (4.0%)	
Fibrous dysplasia	3 (20.0%)	3 (13.0%)	1 (16.7%)	2 (8.0%)	
Osteoid osteoma	1 (6.7%)	1 (4.3%)	1 (16.7%)	2 (8.0%)	
Enchondroma	1 (6.7%)	5 (21.7%)	1 (16.7%)	4 (16.0%)	
Osteochondroma	4 (26.7%)	5 (21.7%)	1 (16.7%)	5 (20.0%)	
Simple bone cyst	1 (6.7%)	1 (4.3%)	0 (0%)	5 (20.0%)	
Others	3 (20.0%)	6 (26.1%)	1 (16.7%)	6 (24.0%)	
Soft tissue	n=69	n=86	n=4	n=99	0.24
Neurofibromatosis	4 (5.8%)	4 (4.7%)	0 (0%)	1 (1.0%)	
Schwannoma	9 (13.0%)	19 (22.1%)	1 (25.0%)	19 (19.2%)	
Lipoma	24 (34.8%)	22 (25.6%)	0 (0%)	39 (39.4%)	
Tenosynovial giant cell tumor	4 (5.8%)	10 (11.6%)	1 (25.0%)	7 (7.1%)	
Fibroma	1 (1.4%)	3 (3.5%)	1 (25.0%)	2 (2.0%)	
Hemangioma	5 (7.2%)	7 (8.1%)	0 (0%)	7 (7.1%)	
Others	22 (31.9%)	21 (24.4%)	1 (25.0%)	24 (24.2%)	

[a] Pearson's χ^2 test and residual analysis.

Bold values indicate significant differences between groups (adjusted standardized residuals >2.58 or <-2.58, which represent p <0.01).

USS/UPS, undifferentiated spindle cell sarcoma/undifferentiated pleomorphic sarcoma.

Supplementary Table 2: Histological diagnosis of participants in the exploratory set

	Primary	Recurrent
Malignant		
Bone		n=5
Ewing sarcoma		1 (20.0%)
Osteosarcoma		4 (80.0%)
Soft tissue		n=98
Myxoid liposarcoma		9 (9.2%)
Myxofibrosarcoma	-	6 (6.1%)
Ewing sarcoma		2 (2.0%)
USS/UPS		13 (13.3%)
Rhabdomyosarcoma		3 (3.1%)
Synovial sarcoma		6 (6.1%)
Dedifferentiated liposarcoma		21 (21.4%)
Others		38 (38.8%)
Intermediate		
Bone	n=48	n=8
Atypical cartilaginous tumor/chondrosarcoma, grade I	7 (14.6%)	2 (25.0%)
Aneurysmal bone cyst	7 (14.6%)	1 (12.5%)
Giant cell tumor of bone	23 (47.9%)	5 (62.5%)
Osteoblastoma	3 (6.3%)	0 (0%)
Chondroblastoma	3 (6.3%)	0 (0%)
Langerhans cell histiocytosis	4 (8.3%)	0 (0%)
Others	1 (2.1%)	0 (0%)
Soft tissue	n=73	n=15
Atypical lipomatous tumor/well-differentiated liposarcoma	38 (52.1%)	13 (86.7%)
Dermatofibrosarcoma protuberans	9 (12.3%)	0 (0%)
Myoepithelioma	2 (2.7%)	0 (0%)
Desmoid-type fibromatosis	15 (20.5%)	2 (13.3%)
Palmar/plantar fibromatosis	2 (2.7%)	0 (0%)
Solitary fibrous tumor	4 (5.5%)	0 (0%)
Others	3 (4.2%)	0 (0%)
Benign		
Bone		n=5
Inflammation		1 (20.0%)
Fibrous dysplasia		1 (20.0%)
Simple bone cyst		1 (20.0%)
Others		2 (40.0%)
	-	
Soft tissue		n=7
Lipoma		2 (28.6%)
Schwannoma		1 (14.3%)
Tenosynovial giant cell tumor		2 (28.6%)
Others		2 (28.6%)

USS/UPS, undifferentiated spindle cell sarcoma/undifferentiated pleomorphic sarcoma.

Supplementary Table 3: Differences in characteristics between the malignant, benign, healthy, and intermediate groups

		Malignant	Benign	Healthy	Intermediate	*P* value
Discovery set		n=77	n=84			
Age (y)		45.9 ± 23.6	44.8 ± 18.4			0.76 [a]
Sex	Men	46 (59.7%)	42 (50.0%)	-	-	0.22 [b]
	Women	31 (40.3%)	42 (50.0%)			
Training set		n=117	n=109	n=150		
Age (y)		50.1 ± 21.7	**43.8 ± 18.4**	51.2 ± 12.2		0.002 [c]
Sex	Men	70 (59.8%)	58 (53.2%)	82 (54.7%)	-	0.56 [b]
	Women	47 (40.2%)	51 (46.8%)	68 (45.3%)		
Training set 2		n=10	n=10			
Age (y)		47.7 ± 29.7	41.0 ± 22.9			0.58 [a]
Sex	Men	4 (40.0%)	6 (60.0%)	-	-	0.37 [b]
	Women	6 (60.0%)	4 (40.0%)			
Validation		n=107	n=124	n=125		
Age (y)		43.8 ± 22.9	44.5 ± 19.1	**51.1 ± 12.1**		0.003 [c]
Sex	Men	**69 (64.5%)**	**52 (41.9%)**	68 (54.4%)	-	0.003 [b]
	Women	**38 (35.5%)**	**72 (58.1%)**	57 (45.6%)		
Exploratory					n=121	
(Primary)					43.0 ± 19.4	
Age (y)					70 (57.9%)	
Sex	Men	-	-	-	51 (42.1%)	-
	Women					
Exploratory		n=103	n=12		n=23	
(Recurrent)		51.3 ± 17.9	40.2 ± 17.6		54.1 ± 17.0	0.08 [c]
Age (y)		60 (58.3%)	7 (58.3%)	-	11 (47.8%)	0.65 [b]
Sex	Men	43 (41.7%)	5 (41.7%)		12 (52.2%)	
	Women					

[a] Student's *t*-test, [b] Pearson's χ^2 test and residual analysis, [c] One-way ANOVA (analysis of variance) and Tukey's post-hoc analysis.

Bold values indicate significant differences between groups (adjusted standardized residuals >2.58 or <-2.58, which represent p <0.01).

Supplementary Table 4: Serum expression levels of selected miRNAs in the discovery set

	Malignant (n=77)	Benign (n=84)	Cross-validation score [a]	Fold change
miR-1237-5p	12.6 ± 0.4	12.2 ± 0.3	0.68	1.3
miR-6787-5p	8.8 ± 0.6	9.2 ± 0.5	0.68	1.3
miR-1915-3p	10.4 ± 0.6	9.9 ± 0.4	0.68	1.4
miR-4730	7.5 ± 0.9	6.9 ± 0.9	0.68	1.5
miR-6794-5p	8.2 ± 0.6	8.5 ± 0.4	0.68	1.3
miR-6741-5p	7.7 ± 0.6	8.0 ± 0.4	0.67	1.3
miR-6869-5p	12.8 ± 0.8	12.4 ± 0.6	0.67	1.4
miR-6782-5p	5.9 ± 0.9	6.5 ± 0.6	0.66	1.5
miR-3178	11.5 ± 0.6	11.2 ± 0.4	0.66	1.3
miR-6893-5p	7.6 ± 0.6	8.0 ± 0.6	0.66	1.3
miR-718	7.2 ± 0.8	6.5 ± 0.7	0.66	1.6
miR-1908-3p	6.9 ± 0.9	6.3 ± 0.7	0.65	1.5
miR-4454	10.4 ± 1.0	10.8 ± 0.8	0.65	1.4
miR-6717-5p	6.7 ± 1.3	7.4 ± 0.9	0.65	1.7
miR-7975	8.4 ± 1.1	8.9 ± 0.8	0.65	1.4
miR-937-5p	8.0 ± 0.5	8.3 ± 0.4	0.65	1.2
miR-328-5p	11.6 ± 0.3	11.8 ± 0.3	0.64	1.1
miR-4634	8.8 ± 0.8	8.2 ± 0.5	0.64	1.5
miR-4763-3p	9.5 ± 0.3	9.7 ± 0.4	0.64	1.1
miR-6756-5p	8.9 ± 0.4	9.2 ± 0.3	0.64	1.2
miR-6861-5p	7.8 ± 0.5	8.1 ± 0.5	0.64	1.3
miR-1273g-3p	8.4 ± 1.0	7.9 ± 0.8	0.63	1.4
miR-128-1-5p	6.3 ± 0.6	6.0 ± 0.7	0.63	1.2
miR-365a-5p	6.9 ± 0.8	7.4 ± 0.6	0.63	1.4
miR-4649-5p	11.4 ± 0.8	11.1 ± 0.7	0.63	1.2
miR-663b	8.9 ± 0.8	8.6 ± 0.8	0.63	1.2
miR-665	7.5 ± 0.7	7.0 ± 0.7	0.63	1.5
miR-6766-5p	6.3 ± 0.8	6.6 ± 0.8	0.63	1.3
miR-6819-5p	8.0 ± 0.4	8.2 ± 0.4	0.63	1.2
miR-6836-3p	8.4 ± 1.1	7.8 ± 0.7	0.63	1.5
miR-6885-5p	11.8 ± 0.8	11.4 ± 0.7	0.63	1.3
miR-1246	7.4 ± 2.0	8.1 ± 1.6	0.63	1.7
miR-1470	6.3 ± 0.9	5.9 ± 0.9	0.63	1.3
miR-4442	9.5 ± 0.4	9.4 ± 0.3	0.63	1.1

miR-4488	13.6 ± 0.4	13.3 ± 0.3	0.63	1.2
miR-6088	12 ± 0.4	11.8 ± 0.4	0.63	1.2
miR-619-5p	7.8 ± 1.4	7.2 ± 1.3	0.63	1.5
miR-6789-5p	9.9 ± 0.6	9.7 ± 0.4	0.63	1.2
miR-6829-5p	6.7 ± 0.7	6.9 ± 0.6	0.63	1.2
miR-762	12.9 ± 0.7	12.7 ± 0.5	0.63	1.2
miR-8059	9.8 ± 1.2	10.4 ± 1.0	0.63	1.6
miR-1909-3p	8.5 ± 0.4	8.3 ± 0.3	0.62	1.2
miR-4286	6.5 ± 0.9	6.9 ± 0.6	0.62	1.3
miR-4492	10.4 ± 0.6	10.2 ± 0.4	0.62	1.2
miR-4516	13.3 ± 0.8	13.1 ± 0.7	0.62	1.2
miR-4674	9.7 ± 0.9	9.1 ± 0.8	0.62	1.5
miR-6087	12.6 ± 0.3	12.5 ± 0.4	0.62	1.1
miR-6768-5p	9.8 ± 0.6	9.4 ± 0.5	0.62	1.3
miR-6784-5p	11.6 ± 0.4	11.4 ± 0.5	0.62	1.2
miR-92a-3p	6.3 ± 1.1	6.8 ± 0.9	0.62	1.4
miR-1193	6.3 ± 0.8	6.7 ± 0.7	0.61	1.3
miR-1247-3p	6.8 ± 0.7	7.1 ± 0.6	0.61	1.3
miR-1260b	9.0 ± 0.8	9.3 ± 0.6	0.61	1.2
miR-3195	8.0 ± 0.9	7.4 ± 0.6	0.61	1.5
miR-3196	12.1 ± 0.4	11.8 ± 0.3	0.61	1.2
miR-4258	10.0 ± 0.8	9.5 ± 0.6	0.61	1.4
miR-4449	6.7 ± 0.9	6.2 ± 0.6	0.61	1.4
miR-4697-5p	8.5 ± 0.5	8.4 ± 0.4	0.61	1.1
miR-4727-3p	5.6 ± 1.1	6.0 ± 1.0	0.61	1.3
miR-4787-5p	13.0 ± 0.6	12.7 ± 0.4	0.61	1.2
miR-6887-5p	6.9 ± 1.0	7.3 ± 0.8	0.61	1.4
miR-8089	6.6 ± 0.7	6.9 ± 0.7	0.61	1.2
miR-1343-5p	9.7 ± 0.3	9.5 ± 0.6	0.61	1.1
miR-4257	6.9 ± 0.7	7.3 ± 0.6	0.61	1.3
miR-4665-3p	6.2 ± 0.9	5.9 ± 0.7	0.61	1.2
miR-4787-3p	6.4 ± 1.1	6.2 ± 0.7	0.61	1.1
miR-6131	9.7 ± 2.8	11.2 ± 1.8	0.61	2.7
miR-6515-5p	5.9 ± 1.1	6.3 ± 1.0	0.61	1.4
miR-7641	6.1 ± 1.3	6.7 ± 1.0	0.61	1.5
miR-7847-3p	7.1 ± 0.9	7.5 ± 0.7	0.61	1.3
miR-92a-2-5p	7.4 ± 0.8	7.1 ± 0.7	0.61	1.2
miR-1292-3p	6.2 ± 0.9	5.7 ± 0.9	0.60	1.4

miR-3652	6.3 ± 0.9	6.6 ± 0.7	0.60	1.2
miR-3665	13.4 ± 0.6	13.2 ± 0.5	0.60	1.1
miR-4281	11.4 ± 0.6	11.3 ± 0.5	0.60	1.1
miR-4433b-3p	8.0 ± 0.4	8.2 ± 0.4	0.60	1.1
miR-4728-5p	7.8 ± 0.6	8.0 ± 0.4	0.60	1.1
miR-4736	6.8 ± 1.0	6.5 ± 0.9	0.60	1.3
miR-4745-5p	12.5 ± 0.7	12.2 ± 0.7	0.60	1.2
miR-658	6.8 ± 0.7	6.4 ± 0.7	0.60	1.3
miR-6775-5p	9.1 ± 0.4	9.3 ± 0.7	0.60	1.1
miR-6805-5p	10.6 ± 0.5	10.4 ± 0.4	0.60	1.2
miR-7113-3p	6.3 ± 0.7	5.9 ± 0.7	0.60	1.3

[a] robust discrimination accuracy calculated by leave-one-out cross-validation.

Supplementary Table 5: Odds ratios for discriminating sarcoma

	Training set		Validation set	
	Univariate analysis	Age- and sex-adjusted analysis	Univariate analysis	Age- and sex-adjusted analysis
vs. benign tumors				
Index VI (positive vs. negative)	152 (53–437)	159 (54–470)	22 (11–42)	23 (12–45)
miR-4736 (per 2-fold increase)	6.7 (4.1–11.1)	6.6 (4.0–10.9)	2.9 (2.1–4.0)	2.9 (2.1–4.0)
miR-6836-3p (per 2-fold increase)	7.4 (4.3–12.7)	9.6 (5.2–17.8)	3.0 (2.2–4.2)	3.1 (2.2–4.3)
miR-4281 (per 2-fold increase)	1.4 (0.9–2.2)	1.7 (1.0–2.8)	1.7 (1.0–2.8)	1.6 (1.0–2.7)
miR-762 (per 2-fold increase)	5.6 (3.0–10.5)	8.1 (4.1–16.1)	5.4 (3.0–9.4)	5.4 (3.0–9.6)
miR-658 (per 2-fold increase)	3.4 (2.2–5.4)	3.3 (2.1–5.3)	2.4 (1.7–3.5)	2.4 (1.6–3.5)
miR-4649-5p (per 2-fold increase)	1.1 (0.8–1.5)	1.1 (0.8–1.5)	1.2 (0.9–1.7)	1.2 (0.9–1.7)
miR-4665-3p (per 2-fold increase)	3.1 (2.0–5.0)	3.41 (2.1–5.5)	2.2 (1.4–3.3)	2.3 (1.5–3.4)
vs. healthy				
Index VI (positive vs. negative)	N.A.	N.A.	109 (44–267)	111 (44–281)
miR-4736 (per 2-fold increase)	350 (41–2978)	458 (41–5118)	7.7 (4.9–12.1)	7.6 (4.8–12.1)
miR-6836-3p (per 2-fold increase)	47 (16–138)	139 (36–537)	5.6 (3.5–8.9)	5.9 (3.7–9.4)
miR-4281 (per 2-fold increase)	47 (18–126)	60 (21–169)	14 (6.7–31)	14 (6.2–30)
miR-762 (per 2-fold increase)	33 (13–87)	44 (16–124)	13 (6.0–29)	12 (5.3–29)
miR-658 (per 2-fold increase)	266 (51–1379)	319 (53–1913)	16 (8.2–30)	16 (8.3–31)
miR-4649-5p (per 2-fold increase)	109 (29–412)	112 (29–427)	9.2 (5.6–15)	9.1 (5.4–15)
miR-4665-3p (per 2-fold increase)	162 (43–607)	481 (94–2463)	22 (11–46)	23 (11–47)

Data are expressed as the odds ratio (95% confidence interval).

N.A., not applicable because no samples were misdiagnosed.

Supplementary Table 6: Information on the miScript PCR primers (QIAGEN)

Gene name	Catalog No.	Assay type	Mature miRNA sequence
hsa-miR-7113-3p	MS00048300	mature miRNA assay	CCTCCCTGCCCGCCTCTCTGCAG
hsa-miR-4281	MS00021336	mature miRNA assay	GGGTCCCGGGGAGGGGGG
hsa-miR-4442	MS00041279	mature miRNA assay	GCCGGACAAGAGGGAGG
hsa-miR-663b	MS00021791	mature miRNA assay	GGTGGCCCGGCCGTGCCTGAGG
hsa-miR-4649-5p	MS00040495	mature miRNA assay	TGGGCGAGGGGTGGGCTCTCAGAG
hsa-miR-6885-5p	MS00048125	mature miRNA assay	AGGGGGGCACTGCGCAAGCAAAGCC
hsa-miR-762	MS00021805	mature miRNA assay	GGGGCTGGGGCCGGGGCCGAGC
hsa-miR-4516	MS00037555	mature miRNA assay	GGGAGAAGGGTCGGGGC
hsa-miR-4449	MS00041216	mature miRNA assay	CGTCCCGGGGCTGCGCGAGGCA
hsa-miR-6789-5p	MS00046970	mature miRNA assay	GTAGGGGCGTCCCGGGCGCGCGGG
hsa-miR-4634	MS00043771	mature miRNA assay	CGGCGCGACCGGCCCGGGG
hsa-miR-6836-3p	MS00047544	mature miRNA assay	ATGCCTCCCCCGGCCCCGCAG
hsa-miR-4258	MS00021175	mature miRNA assay	CCCCGCCACCGCCTTGG
hsa-miR-4787-3p	MS00081479	mature miRNA assay	GATGCGCCGCCCACTGCCCCGCGC
hsa-miR-1470	MS00020342	mature miRNA assay	GCCCTCCGCCCGTGCACCCCG
hsa-miR-3195	MS00044975	mature miRNA assay	CGCGCCGGGCCCGGGTT
hsa-miR-718	MS00037856	mature miRNA assay	CTTCCGCCCCGCCGGGCGTCG
hsa-miR-4665-3p	MS00040313	mature miRNA assay	CTCGGCCGCGGCGCGTAGCCCCCGCC
hsa-miR-619-5p	MS00046032	mature miRNA assay	GCTGGGATTACAGGCATGAGCC
hsa-miR-1273g-3p	MSC0076466	mature miRNA assay	ACCACTGCACTCCAGCCTGAG
hsa-miR-665	MS00010493	mature miRNA assay	ACCAGGAGGCTGAGGCCCCT
hsa-miR-4745-5p	MS00045059	mature miRNA assay	TGAGTGGGGCTCCCGGGACGGCG
hsa-miR-658	MS00005369	mature miRNA assay	GGCGGAGGGAAGTAGGTCCGTTGGT
hsa-miR-4736	MS00039606	mature miRNA assay	AGGCAGGTTATCTGGGCTG
hsa-miR-4697-5p	MS00040012	mature miRNA assay	AGGGGGCGCAGTCACTGACGTG
hsa-miR-4727-3p	MS00039690	mature miRNA assay	ATAGTGGGAAGCTGGCAGATTC
hsa-miR-8059	MS00048685	mature miRNA assay	GGGGAACTGTAGATGAAAAGGC
hsa-miR-6087	MS00045402	mature miRNA assay	TGAGGCGGGGGGGCGAGC
hsa-miR-149-3p	MS00037702	mature miRNA assay	AGGGAGGGACGGGGGCTGTGC
hsa-miR-2861	MS00042140	mature miRNA assay	GGGGCCTGGCGGTGGGCGG
hsa-miR-4463	MS00044996	mature miRNA assay	GAGACTGGGGTGGGGCC

Supplementary Methods

RNA extraction from fresh-frozen samples and quality controls

Fresh-frozen tissues were crushed to powder using a Multi-beads Shocker (Yasui Kikai, Osaka, Japan) under cooling with liquid nitrogen. Total RNA was extracted from frozen tumor tissue powder using the miRNeasy Mini Kit (Qiagen, Hilden, Germany). The quality of total RNA was assessed on a 2100 Bioanalyzer (Agilent Technologies, Santa Clara, CA, USA).

MiRNA expression analysis of fresh-frozen samples

Comprehensive miRNA expression analysis was performed on 250 ng of total RNAs from fresh-frozen samples using the 3D-Gene® miRNA Labeling kit and the 3D-Gene® Human miRNA Oligo Chip (Toray Industries, Inc., Tokyo, Japan), which was designed to detect 2565 miRNA sequences registered in miRBase release 21 (http://www.mirbase.org/). For quality control of microarray data, a coefficient of variation for negative control probes >0.15 or more than 10 flagged probes was considered evidence of low-quality results, and samples meeting either criterion were excluded from further analyses. A miRNA was considered to be present if the corresponding signal was greater than the mean + 2 × standard deviations of the negative control signal (from which the top and bottom 5%, ranked by signal intensity, had been removed). Once a miRNA was considered present, the mean signal of the negative controls (from which the top and bottom 5%, ranked by signal intensity, had been removed) was subtracted from the miRNA signal. When the signal value was negative (or undetected) after background subtraction, the value was replaced by the lowest signal intensity on the microarray minus 0.1 on a base-2 logarithmic scale. A global median method was used to normalize signals among microarrays.

Algorithm: combinatorial optimization for multi-candidate miRNAs

Notation:

N: number of candidate sets of miRNAs desired.

M: maximum combination number of miRNAs of each candidate set desired.

Score: Accuracy = (TP + TN) / (TP + FP + FN + TN); each abbreviation is defined below.

	cancer	no cancer
prediction positive	TP: number of true positives	FP: number of false positives
prediction negative	FN: number of false negatives	TN: number of true negatives

Step 1: Set N and M.

Step 2: FOR EACH miRNA:

Evaluate the average score (Accuracy) for a selected miRNA by means of linear discriminant analysis with leave-one-out (LOO) cross-validation.

Step 3: Sort the result in descending order, and keep the top N miRNAs as a first candidate set for the next step.

Step 4: FOR i = 2 TO M:

Step 4-1: FOR EACH of N candidates:

Make all combinations of the remaining miRNAs against the selected candidate (set) and obtain scores by means of linear discriminant analysis with LOO (same as step 2).

Step 4-2: Sort scores for all combinations [N × (total number of miRNAs – i)] and select the top N sets of combinations consist of i miRNAs in the next step.
