# Supplementary material for: A serum microRNA classifier for the diagnosis of sarcomas of various histological subtypes
Source: Nat Commun. 2019 Mar 21;10:1299. doi: 10.1038/s41467-019-09143-8 (PMC6428849; doi:10.1038/s41467-019-09143-8)
Supplement: Supplementary file 3 — Reporting Summary [file 41467_2019_9143_MOESM3_ESM.pdf]

nature research

Corresponding author(s): Takahiro Ochiya

Last updated by author(s): Feb 7, 2019

Reporting Summary

Nature Research wishes to improve the reproducibility of the work that we publish. This form provides structure for consistency and transparency in reporting. For further information on Nature Research policies, see Authors & Referees and the Editorial Policy Checklist.

Statistics

For all statistical analyses, confirm that the following items are present in the figure legend, table legend, main text, or Methods section.

n/a	Confirmed	
☐	☒	The exact sample size (*n*) for each experimental group/condition, given as a discrete number and unit of measurement
☐	☒	A statement on whether measurements were taken from distinct samples or whether the same sample was measured repeatedly
☐	☒	The statistical test(s) used AND whether they are one- or two-sided<br>*Only common tests should be described solely by name; describe more complex techniques in the Methods section.*
☐	☒	A description of all covariates tested
☐	☒	A description of any assumptions or corrections, such as tests of normality and adjustment for multiple comparisons
☐	☒	A full description of the statistical parameters including central tendency (e.g. means) or other basic estimates (e.g. regression coefficient) AND variation (e.g. standard deviation) or associated estimates of uncertainty (e.g. confidence intervals)
☐	☒	For null hypothesis testing, the test statistic (e.g. *F*, *t*, *r*) with confidence intervals, effect sizes, degrees of freedom and *P* value noted<br>*Give P values as exact values whenever suitable.*
☒	☐	For Bayesian analysis, information on the choice of priors and Markov chain Monte Carlo settings
☐	☒	For hierarchical and complex designs, identification of the appropriate level for tests and full reporting of outcomes
☐	☒	Estimates of effect sizes (e.g. Cohen's *d*, Pearson's *r*), indicating how they were calculated

Our web collection on statistics for biologists contains articles on many of the points above.

Software and code

Policy information about availability of computer code

Data collection	Not applicable
Data analysis	Discrimination analyses were performed using R version 3.1.2 (R Foundation for Statistical Computing, http://www.R-project.org), compute.es package version 0.2-4, glmnet package version 2.0-3, hash package version 2.2.6, MASS package version 7.3-45, mutoss package version 0.1-10, and pROC package version 1.8. Unsupervised average linkage clustering, heatmap generation with Euclidean distances, and PCA were performed using Partek Genomics Suite 6.6 (Partek, St. Louis, MO, USA). Other statistical analyses, including logistic regression analysis, were performed using IBM SPSS Statistics version 22 (IBM Japan, Tokyo, Japan).

For manuscripts utilizing custom algorithms or software that are central to the research but not yet described in published literature, software must be made available to editors/reviewers. We strongly encourage code deposition in a community repository (e.g. GitHub). See the Nature Research guidelines for submitting code & software for further information.

Data

Policy information about availability of data

All manuscripts must include a data availability statement. This statement should provide the following information, where applicable:

- Accession codes, unique identifiers, or web links for publicly available datasets
- A list of figures that have associated raw data
- A description of any restrictions on data availability

All miRNA microarray data and clinical information on the patients who provided the serum samples have been deposited in the Gene Expression Omnibus (GEO) (https://www.ncbi.nlm.nih.gov/geo/) database (accession number: GSE124158). All other relevant data are available within the article file or Supplementary Information, or available from the authors on reasonable request.

Field-specific reporting

Please select the one below that is the best fit for your research. If you are not sure, read the appropriate sections before making your selection.

☒ Life sciences ☐ Behavioural & social sciences ☐ Ecological, evolutionary & environmental sciences

For a reference copy of the document with all sections, see nature.com/documents/nr-reporting-summary-flat.pdf

Life sciences study design

All studies must disclose on these points even when the disclosure is negative.

Sample size	We obtained 1002 serum samples derived from patients with bone and soft tissue tumors. After excluding five patients with uterine sarcoma, 24 patients who had undergone treatment before serum collection, and 76 low-quality samples, we analyzed the miRNA profiles in material from 897 patients, including serum samples from patients with malignant bone and soft tissue tumors (sarcomas) (n=414), intermediate tumors (n=144), and benign or non-tumors (n=339). Samples from patients with sarcomas and benign tumors were divided into four cohorts: discovery, training, and validation sets for microarray analysis; and training set 2 for quantitative RT-PCR. Serum samples from 150 and 125 healthy volunteers were included in the training and validation cohorts, respectively. Because the samples were chronologically divided into three cohorts, the sample size for each cohort could not be determined based on a pre-specified effect size.
Data exclusions	Patients with uterine sarcoma (n=5) and those treated with chemotherapy or radiotherapy before serum collection (n=24) were excluded, as were patients with poor-quality microarray data (n=76).
Replication	The reproducibility of the microarray analysis was confirmed by performing microarray analysis on the same RNA sample for fifteen times. A strong correlation between the fifteen replicates was indicated (Pearson's correlation coefficient [R], 0.96 [95% confidence interval, 0.94-0.98])
Randomization	The training and validation cohorts were randomly divided using computer-generated random numbers.
Blinding	The collection of clinical information and miRNA expression analysis were blindly performed by different members.

Reporting for specific materials, systems and methods

We require information from authors about some types of materials, experimental systems and methods used in many studies. Here, indicate whether each material, system or method listed is relevant to your study. If you are not sure if a list item applies to your research, read the appropriate section before selecting a response.

Materials & experimental systems

n/a	Involved in the study	
☒	☐	Antibodies
☒	☐	Eukaryotic cell lines
☒	☐	Palaeontology
☒	☐	Animals and other organisms
☐	☒	Human research participants
☒	☐	Clinical data

Methods

n/a	Involved in the study	
☒	☐	ChIP-seq
☒	☐	Flow cytometry
☒	☐	MRI-based neuroimaging

Human research participants

Policy information about studies involving human research participants

Population characteristics	Clinical features, including age, sex, site of primary tumor, and TNM stage, did not differ significantly between the sets (P > 0.01 after Bonferroni correction for multiple testing). The distribution of histological subtypes of bone and soft tissue tumors is shown in Supplemental Tables 1 and 2. Age and sex distributions of malignant, benign, and healthy participants were matched in the discovery set, but not in the training and validation sets (Supplemental Table 3). Therefore, age- and sex-adjusted analysis was conducted after the identification of a diagnostic index, as described below.
Recruitment	Serum samples were obtained consecutively from 1002 patients with bone and soft tissue tumors who were admitted or referred to the The National Cancer Center Hospital (NCCH) between 2007 and 2013. Non-tumor serum samples were obtained from healthy volunteers aged >35 years who were recruited from the Yokohama Minoru Clinic in 2015.
Ethics oversight	This study was approved by the NCCH Institutional Review Board (2004-050, 2013-111, 2015-266) and the Research Ethics Committee of Medical Corporation Shintokai Yokohama Minoru Clinic (6019-18-3772).

Note that full information on the approval of the study protocol must also be provided in the manuscript.
